# The mediating effect of family resilience between readiness for hospital discharge and self-management efficacy among breast cancer patients

**DOI:** 10.3389/fpsyg.2025.1716566

**Published:** 2025-12-17

**Authors:** Shan Wang, Haochan Wu, Hongkun Xu, Liyan Zhou, Dewu Xu, Xiaoyun Qin, Ling Chen

**Affiliations:** 1 Department of Operating Room, Affiliated Hospital of Jiangnan University, Wuxi, Jiangsu, China; 2 Obstetrics, Gynecology and Reproduction Research, Affiliated Hospital of Jiangnan University, Wuxi, Jiangsu, China; 3 Department of Medical Education, Affiliated Hospital of Jiangnan University, Wuxi, Jiangsu, China; 4 Department of Breast Surgery, Affiliated Hospital of Jiangnan University, Wuxi, Jiangsu, China

**Keywords:** breast cancer, readiness for hospital discharge, family resilience, self-management efficacy, mediation

## Abstract

**Background:**

Readiness for hospital discharge is a critical factor in the recovery of breast cancer patients, facilitating the transition from inpatient care to home-based self-management. While adequate preparation equips patients with the necessary knowledge and skills, effective behavioral change often requires support from the family system. Grounded in Family Systems Theory and Family Resilience Model, this study examined the relationship between readiness for hospital discharge (RHD) and self-management efficacy (SME) and tested the mediating role of family resilience (FR)—a key indicator of family adaptability.

**Methods:**

A cross-sectional survey was conducted between February 2024 and January 2025 at a tertiary hospital in Wuxi, China. Data were collected from 265 postoperative breast cancer patients using the Readiness for Hospital Discharge Scale (RHDS), the Family Hardiness Index (FHI), and the Self-Management Efficacy Scale for Cancer Patients (C-SUPPH). Pearson correlation analysis and structural equation modeling (SEM) were employed to examine associations and mediation effects.

**Results:**

Of the 274 questionnaires distributed, 265 were valid (response rate: 96.72%). The mean scores were 85.56 ± 9.62 (RHDS), 42.55 ± 5.70 (FHI), and 95.62 ± 13.16 (C-SUPPH). Readiness for hospital discharge was significantly correlated with both family resilience (*r* = 0.343, *p* < 0.01) and self-management efficacy (*r* = 0.394, *p* < 0.01). SEM results indicated that family resilience partially mediated the relationship between Readiness for hospital discharge and self-management efficacy, accounting for 29.60% of the total effect.

**Conclusion:**

Readiness for hospital discharge significantly predicts self-management efficacy in breast cancer patients, with family resilience serving as a key mediating factor. These findings support targeted interventions to improve discharge preparation and family resilience, thereby enhancing recovery and outcomes.

## 1 Introduction

According to data released by the International Agency for Research on Cancer (IARC) in 2022, there were approximately 2.309 million new cases of breast cancer (BC) globally and about 666,000 related deaths, ranking it second in incidence and fourth in mortality among all cancers (Bray et al., 2024). Notably, China accounted for 21.11% of the global new cases and 11.96% of the deaths, highlighting the significant burden of BC in the country (Han et al., 2024). A comprehensive treatment strategy usually entails collaboration across multiple disciplines, incorporating surgical intervention, chemotherapy, radiotherapy, hormonal therapy, and molecularly targeted treatments (Zeng et al., 2018). While these treatments have significantly improved survival rates (Miller et al., 2022), they also impose long-term physiological burdens and psychological distress (Singh et al., 2022; Jones et al., 2015). Following initial treatment, patients often undergo a critical transition from hospital-based care to home-based recovery (Kim et al., 2024), and the success of this transition largely depends on the establishment and maintenance of self-management capacity (Cheng et al., 2017).

Self-management efficacy (SME) is a refined and context-specific extension of the broader concept of self-efficacy in chronic illness management (Bandura, 1977). It reflects a patient’s confidence in performing health-related behaviors—such as medication adherence, dietary control, physical activity, symptom monitoring, and follow-up attendance—and serves as a psychological foundation for sustained behavioral engagement (Chou, 2019). Evidence consistently shows that higher SME enhances treatment adherence, promotes rehabilitation behaviors, and mitigates psychological stress through improved perceived control, thereby reducing anxiety and depression and improving quality of life (Singleton et al., 2022). In BC populations, SME is especially critical: patients with strong self-management efficacy are more likely to actively cope with treatment side effects, maintain healthy lifestyles, and achieve better functional recovery (Kim et al., 2025). Conversely, low SME is often linked to passive coping, higher dependence, and poorer long-term health outcomes (Wang et al., 2017). Therefore, enhancing SME has become a central focus of patient-centered cancer rehabilitation and survivorship care.

In the context of cancer survivorship, family resilience (FR) plays a pivotal role in promoting psychological adaptation and sustained self-management behaviors. Originating from the Family Resilience Theory proposed by McCubbin et al. (1996), FR refers to a family system’s ability to withstand, adapt to, and recover from adversity. Walsh later conceptualized FR as encompassing three interrelated domains: (1) a shared belief system that fosters meaning-making and a positive outlook, (2) organizational patterns that promote flexibility, mutual support, and resource mobilization, and (3) communication and problem-solving processes that enable emotional expression and collaborative decision-making (Walsh, 1996). Empirical studies have shown that families with high resilience can integrate illness into daily life, maintain hope, and mobilize internal and external resources to face ongoing challenges, thereby reducing stress and enhancing adaptive functioning in chronic illness contexts (Shao et al., 2025). In contrast, families with low resilience tend to exhibit emotional disengagement, poor communication, and limited coping capacity, which may exacerbate patients’ distress and hinder recovery (He et al., 2024). Thus, family resilience functions as a crucial psychosocial resource that supports patients’ long-term self-management and well-being.

Readiness for Hospital Discharge (RHD), first introduced by Fenwick (1979), refers to a comprehensive assessment of whether a patient is adequately prepared to transition safely from inpatient care to home and community life. It encompasses physical, psychological, and social dimensions of preparedness. Later, Galvin expanded this concept by emphasizing RHD as a dynamic process, reflecting patients’ subjective perceptions of their health status, disease knowledge, caregiving resources, and social support before discharge (Galvin et al., 2017). Studies have found that higher levels of RHD are associated with better disease understanding, greater coping capacity, and enhanced self-management confidence, leading to smoother recovery and fewer complications after discharge (Wen et al., 2024). Conversely, when social and family support is lacking, the benefits of discharge education may fail to translate into sustained behavioral change (Huang et al., 2022).

In BC rehabilitation, where recovery often relies heavily on family caregiving, the family environment and its adaptive capacity are essential in bridging hospital-based care with home-based recovery (Cui et al., 2023).

This study is grounded in Family Systems Theory (FST) (Bowen, 1993) and Family Resilience Model (FRM) (Walsh, 1996), which together offer a comprehensive framework for understanding how intra-family dynamics influence individual health behaviors. According to FST, the family functions as an interdependent emotional unit in which changes in one member influence the entire system. Thus, an individual’s readiness for discharge can serve as a catalyst that stimulates adaptive family responses—such as increased communication, coordination, and emotional support—thereby reinforcing family resilience. FRM further explains how families mobilize internal and external resources to maintain cohesion and promote recovery under stress. Within this framework, family resilience operates as a dynamic process that enables families to support individual well-being through shared belief systems, flexible organization, and constructive communication. Integrating these perspectives, this study proposes a systemic and dynamic pathway in which RHD activates family resilience, which in turn enhances SME. This conceptualization emphasizes the reciprocal relationship between individual and family subsystems: improved discharge readiness facilitates family adaptive functioning, while resilient family processes create an environment that reinforces self-management confidence and behaviors.

**Figure 1:**
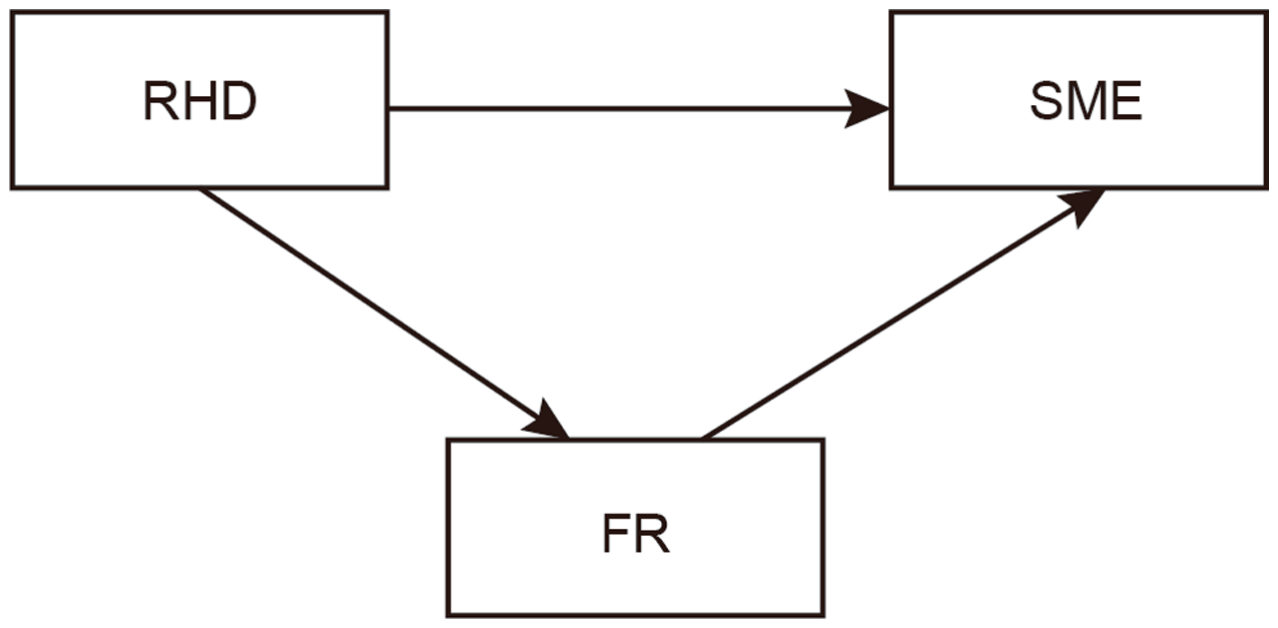
The hypothesis model of readiness for hospital discharge (RHD), family resilience (FR), and self-management efficacy (SME) in patients with breast cancer.

Based on this framework, a structural equation modeling (SEM) was constructed to examine the following hypotheses (see Figure 1):

*H1*: *There are significant correlations among readiness for hospital discharge, family resilience, and self-management efficacy: readiness for hospital discharge is positively associated with family resilience, which in turn is positively associated with self-management efficacy.*

*H2*: *Family resilience mediates the relationship between readiness for hospital discharge and self-management efficacy: higher readiness for hospital discharge enhances perceived family resilience, which subsequently improves self-management efficacy.*

Findings from this study may help elucidate behavioral mechanisms underlying the discharge-to-recovery trajectory among BC patients and provide both theoretical and empirical support for family-centered nursing interventions.

## 2 Materials and methods

### 2.1 Ethical approval

This study was approved by the Medical Ethics Committee of the Affiliated Hospital of Jiangnan University (Approval No. LS2023072). Written informed consent was obtained from all participants, and patient privacy was protected throughout the entire research process.

### 2.2 Study design and participants

This cross-sectional survey was conducted using a convenience sampling method in the Department of Breast Surgery, Affiliated Hospital of Jiangnan University, between February 2024 and January 2025. The inclusion criteria were as follows: (1) age ≥18 years; (2) pathologically confirmed BC; (3) had undergone surgical treatment and were in the postoperative recovery phase; (4) clinically stable and ready for discharge; (5) adequate reading and comprehension ability; (6) willingness to participate with informed consent. Exclusion criteria included: (1) diagnosis of other malignancies or severe organic diseases; (2) a history of psychiatric or cognitive disorders, or currently receiving psychological treatment.

The sample size was determined using an SEM sample size calculator (Soper, 2018). With an anticipated effect size of 0.30, a desired statistical power level of 0.95, and a significance level (*α*) of 0.05, the model included three latent variables and nine observed variables. According to the calculator’s recommendation, the minimum sample size for model structure was 200. Considering a potential dropout rate of approximately 20%, the final estimated sample size was 250. Ultimately, 265 breast cancer patients were enrolled, exceeding the minimum requirement for adequate statistical power.

### 2.3 Data collection

Before the formal survey, the researchers explained the study objectives, significance, and questionnaire requirements to all participants. Data collection was conducted by a team of three investigators who had received standardized training to ensure consistency of terminology and a uniform understanding of the measurement scales.

To minimize potential biases during data collection, several measures were taken. First, a standardized protocol and unified instructions were used to reduce interviewer and procedural bias. Second, participants were recruited consecutively from the same hospital during the study period to reduce selection bias. Third, all participants completed the questionnaires independently in a quiet setting, and investigators provided neutral clarifications only when necessary to prevent information bias. Finally, completed questionnaires were checked on-site for completeness to minimize missing data.

**Figure 2:**
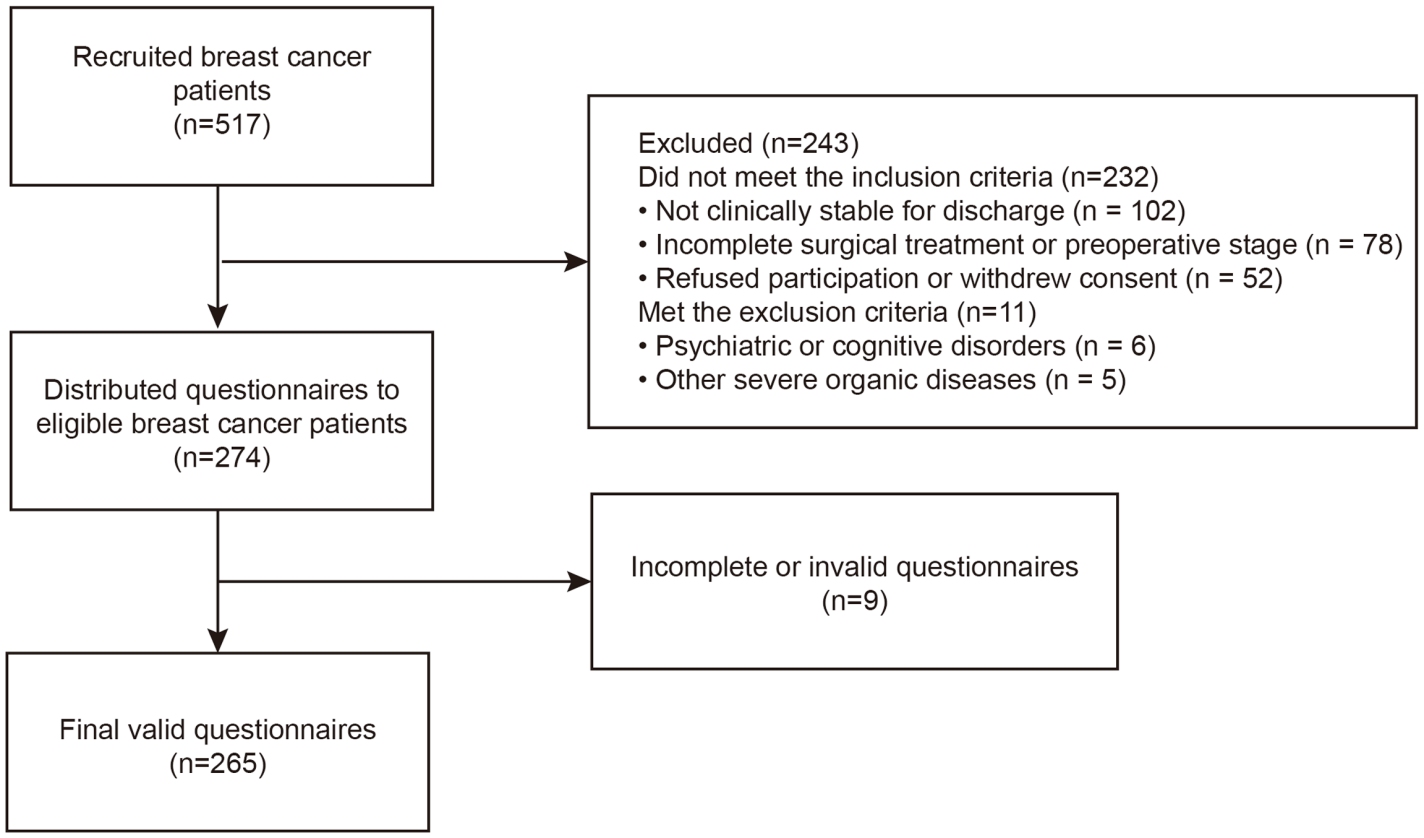
Flow diagram of participant recruitment (breast cancer patients).

Participation was entirely voluntary, and participants were assured of anonymity and the right to withdraw at any time. All privacy and confidentiality requirements were strictly observed. A total of 274 questionnaires were distributed, with 265 valid responses returned, yielding an effective response rate of 96.72%. The recruitment process is shown in Figure 2.

### 2.4 Instrument

#### 2.4.1 General demographic information questionnaire

The questionnaire was developed by the researchers based on a review of relevant literature. It consisted of two sections: demographic characteristics and clinical information. The items included age, education level, religious belief, occupation, monthly household income, place of residence, length of marriage, marital quality, parental status, TNM stage, surgical method, and current treatment.

#### 2.4.2 Readiness for Hospital Discharge Scale

The Readiness for Hospital Discharge Scale (RHDS) was originally developed by Weiss and Piacentine (2006), guided by Meleis’ Transition Theory, to evaluate patients’ readiness for hospital discharge. A simplified Chinese version was adapted by Lin et al. (2014), consisting of 12 items across three dimensions: personal status, coping ability, and expected support. Each item is rated on a scale from 0 to 10, with a total score ranging from 0 to 120. Higher scores indicate better Readiness for hospital discharge. A score below 7 suggests inadequate readiness, 7–8 indicates moderate readiness, 8–9 reflects a relatively high level, and above 9 represents high readiness. The Chinese version has been validated among BC patients, demonstrating good reliability and construct validity (Zhu et al., 2025). In this study, the Cronbach’s *α* coefficient of the RHDS was 0.864, indicating good internal consistency.

#### 2.4.3 Family Hardiness Index

The Family Hardiness Index (FHI) was originally developed by McCubbin et al. in 1986 to assess internal family strengths in the face of stress and adversity. A Chinese version of the scale was later adapted by Taiwanese scholar Su-Chun Kuo, who modified the items to better fit the cultural characteristics of Chinese populations. The scale consists of 20 items divided into three dimensions: commitment, control, and challenge. A 4-point Likert scale is used, with response options ranging from “completely false” to “completely true,” scored from 0 to 3. Among the items, 11 are positively worded and 9 are reverse-scored. The total score ranges from 0 to 60, with higher scores indicating greater family resilience. The FHI has been applied in studies involving BC patients and their family caregivers in China, showing satisfactory psychometric properties and high internal consistency (Huang et al., 2019; Sun et al., 2024). In this study, the Cronbach’s *α* coefficient of the FHI was 0.875, indicating good internal consistency.

#### 2.4.4 Self-Management Efficacy Scale for Cancer Patients

The Strategies Used by People to Promote Health scale (SUPPH) was originally developed by Lev and Owen (1996) and was adapted into Chinese by Qian and Yuan in 2011 (Zhuang et al., 2024), renamed the Chinese version of the Self-Management Efficacy Scale for Cancer Patients (C-SUPPH). The scale consists of 28 items divided into three dimensions: stress reduction, positive attitude, and self-decision-making. Items are rated on a five-point Likert scale, with a total score range of 28 to 140 points. A higher score indicates stronger self-management efficacy. Scores ≤65 reflect low efficacy, 66–102 represent moderate efficacy, and ≥103 indicate high efficacy. The C-SUPPH has been widely used and validated in BC populations in China, demonstrating excellent reliability and construct validity (Zhuang et al., 2024). In this study, the Cronbach’s α coefficient of the C-SUPPH was 0.941, indicating good internal consistency.

### 2.5 Statistical analysis

All statistical analyses were performed using SPSS version 26.0 and AMOS version 26.0. Before analysis, the distribution of all continuous variables was assessed using the Shapiro–Wilk test, Q–Q plots, and skewness–kurtosis coefficients. Continuous variables with a normal distribution were expressed as mean ± standard deviation (SD), and categorical variables were presented as frequencies and percentages [n (%)]. Independent sample t-tests or one-way ANOVA were used to compare differences between groups. Pearson correlation analysis was conducted to examine the associations among readiness for hospital discharge, family resilience, and self-management efficacy. An SEM was constructed using AMOS 26.0 to explore the mediating effect of family resilience between readiness for hospital discharge (independent variable) and self-management efficacy (dependent variable). Bootstrapping with 5,000 resamples was used to test the significance of the mediating effect. A 95% bias-corrected confidence interval (CI) that did not include zero indicated a significant mediation effect. Model fit was evaluated using the following indices: the chi-square to degrees of freedom ratio (χ^2^/df), root mean square error of approximation (RMSEA), goodness-of-fit index (GFI), adjusted goodness-of-fit index (AGFI), comparative fit index (CFI), Tucker–Lewis index (TLI), normed fit index (NFI), and incremental fit index (IFI). A two-sided *p*-value < 0.05 was considered statistically significant.

## 3 Results

### 3.1 Socio-demographic characteristics of participants

A total of 265 BC patients aged 26 to 68 years (mean age: 50.99 ± 9.45 years) were included in this study. Among them, 36.2% had completed senior high school or vocational education, and 93.2% reported no religious affiliation. Regarding employment status, 68.3% were retired or unemployed, and 38.5% resided in urban areas. In terms of marital and family characteristics, 98.1% were married, and 94.0% had children. Clinically, 82 patients (30.9%) were diagnosed with stage I BC, 124 (46.8%) with stage II, and 59 (22.3%) with stage III. Breast-conserving surgery was performed in 34.3% of patients, and 68.7% were undergoing postoperative adjuvant chemotherapy at the time of the survey. Statistically significant differences were found between groups based on TNM stage and type of surgery (*p* < 0.05; Table 1).

**Table 1:** Univariate analysis of self-management efficacy in breast cancer patients (*N* = 265).

Characteristic	*n* (%)	Self-management efficacy (Mean ± SD)	t/F	*p*
Age (years)			2.043	0.132
<40	29 (10.9)	98.90 ± 12.33		
40–59	181 (68.3)	95.88 ± 12.41		
≥60	55 (20.8)	93.00 ± 15.52		
Education level			1.295	0.276
Junior high school or below	94 (35.5)	93.96 ± 14.16		
Senior high school/vocational school	96 (36.2)	96.06 ± 12.85		
College/bachelor’s degree	75 (28.3)	97.12 ± 12.14		
Religious belief			−0.242	0.809
Yes	18 (6.8)	95.67 ± 13.06		
No	247 (93.2)	94.89 ± 14.78		
Occupation			0.735	0.463
Incumbency	84 (31.7)	96.49 ± 12.08		
Unemployed/ Retired	181 (68.3)	95.21 ± 13.64		
Monthly household income			2.266	0.106
<3,000	68 (25.7)	93.35 ± 14.41		
3,000–4,999	94 (35.5)	95.09 ± 12.95		
≥5,000	103 (38.9)	97.59 ± 12.29		
Place of residence			1.937	0.146
Rural area	67 (25.3)	92.99 ± 13.51		
County district	96 (36.2)	96.01 ± 13.94		
Urban area	102 (38.5)	96.97 ± 11.99		
Marriage			0.208	0.835
Yes	260 (98.1)	95.64 ± 13.17		
No	5 (1.9)	94.40 ± 14.03		
Parental status			−0.297	0.767
Yes	249 (94.0)	95.55 ± 13.36		
No	16 (6.0)	96.56 ± 9.61		
TNM stage			5.425	0.005
I	82 (30.9)	99.43 ± 11.52		
II	124 (46.8)	94.40 ± 14.30		
III	59 (22.3)	92.88 ± 11.73		
Surgical method			5.557	0.004
Breast-conserving surgery	91 (34.3)	99.23 ± 11.68		
Total mastectomy	81 (30.6)	94.28 ± 12.49		
Modified radical mastectomy	93 (35.1)	93.24 ± 14.40		
Current adjuvant therapy			−1.119	0.264
None	83 (31.3)	94.28 ± 13.29		
Postoperative chemotherapy	182 (68.7)	96.23 ± 13.09		

### 3.2 Mean scores for RHDS, FHI, and C-SUPPH among BC patients

The mean scores for RHDS, FHI, and C-SUPPH among BC patients were 85.56 ± 9.62, 42.55 ± 5.70, and 95.62 ± 13.16, respectively. The number of dimensions and score ranges for each scale are presented in Table 2.

**Table 2:** Specific descriptive analysis of readiness for hospital discharge, family resilience, and self-management efficacy scores (*N* = 265).

Variables	Items	Score range	Mean ± SD
RHDS	12	62 ~ 109	85.56 ± 9.62
Personal status	3	10 ~ 27	19.00 ± 3.12
Coping ability	5	25 ~ 49	38.02 ± 4.53
Expected support	4	20 ~ 40	28.58 ± 3.47
FHI	15	31 ~ 60	42.55 ± 5.70
Commitment	9	14 ~ 27	22.04 ± 2.76
Control	6	6 ~ 18	10.68 ± 2.41
Challenge	5	6 ~ 15	9.98 ± 1.79
C-SUPPH	28	65 ~ 132	95.62 ± 13.16
Positive attitude	15	34 ~ 71	51.47 ± 7.44
Self-decompression	10	18 ~ 48	34.17 ± 6.74
Self-decision-making	3	5 ~ 15	9.98 ± 2.43

RHDS, Readiness for Hospital Discharge Scale; FHI, Family Hardiness Index; C-SUPPH, Self-Management Efficacy for Cancer Patients.

### 3.3 Correlation analysis between RHDS, FHI, and C-SUPPH in BC patients

Pearson correlation analysis demonstrated that RHDS was positively associated with FHI (*r* = 0.343, *p* < 0.01) and positively related to C-SUPPH (*r* = 0.394, *p* < 0.01). Moreover, FHI showed a significant positive correlation with C-SUPPH (*r* = 0.391, *p* < 0.01). The detailed correlation coefficients are presented in Table 3.

**Table 3:** Pearson correlation analysis of readiness for hospital discharge, family resilience, and self-management efficacy among breast cancer patients (*N* = 265).

Variables	1	2	3	4	5	6	7	8	9	10	11	12
1. FHI	1.000											
2. Commitment	0.892^**^	1.000										
3. Control	0.897^**^	0.720^**^	1.000									
4. Challenge	0.695^**^	0.417^**^	0.493^**^	1.000								
5. RHDS	0.343^**^	0.298^**^	0.299^**^	0.274^**^	1.000							
6. Personal status	0.300^**^	0.226^**^	0.246^**^	0.316^**^	0.837^**^	1.000						
7. Coping ability	0.290^**^	0.275^**^	0.254^**^	0.196^**^	0.906^**^	0.635^**^	1.000					
8. Expected support	0.297^**^	0.259^**^	0.273^**^	0.214^**^	0.848^**^	0.600^**^	0.651^**^	1.000				
9. C-SUPPH	0.391^**^	0.361^**^	0.379^**^	0.262^**^	0.394^**^	0.380^**^	0.309^**^	0.355^**^	1.000			
10. Positive attitude	0.300^**^	0.273^**^	0.301^**^	0.200^**^	0.332^**^	0.365^**^	0.246^**^	0.279^**^	0.838^**^	1.000		
11. Self-decompression	0.345^**^	0.312^**^	0.323^**^	0.244^**^	0.342^**^	0.290^**^	0.292^**^	0.315^**^	0.823^**^	0.420^**^	1.000	
12. Self-decision-making	0.243^**^	0.253^**^	0.237^**^	0.129^**^	0.168^**^	0.134^**^	0.112	0.196^**^	0.565^**^	0.312^**^	0.399^**^	1.000

**p < 0.01.

RHDS, Readiness for Hospital Discharge Scale; FHI, Family Hardiness Index; C-SUPPH, Self-Management Efficacy for Cancer Patients.

**Figure 3:**
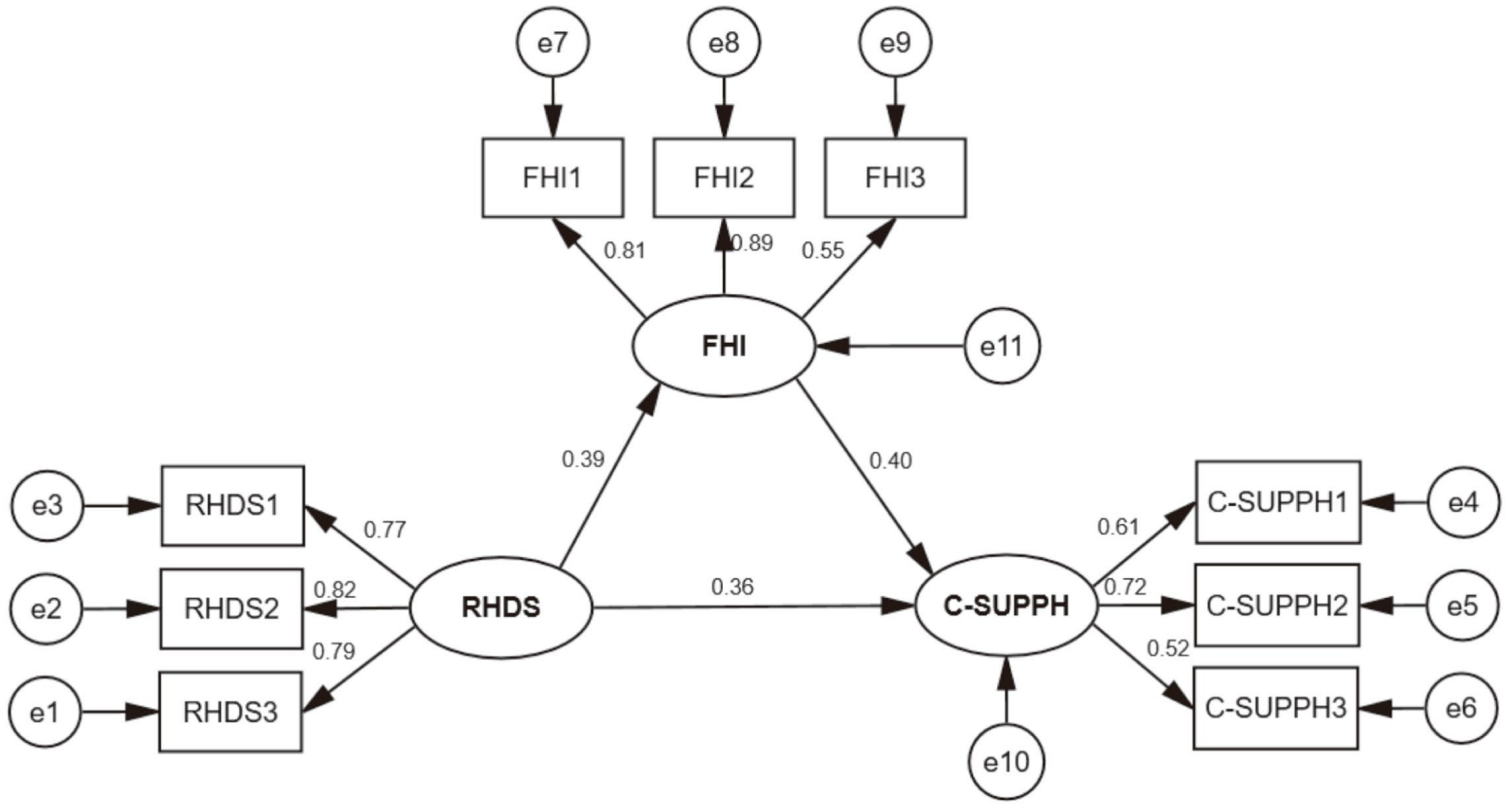
The structural equation modeling of readiness for hospital discharge (RHD), family resilience (FR), and self-management efficacy (SME). RHDS: Readiness for Hospital Discharge Scale. RHDS1: Personal status. RHDS2: Coping ability. RHDS3: Expected support. FHI: Family Hardiness Index. FHI1: Commitment. FHI2: Control. FHI3: Challenge. C-SUPPH: Self-Management Efficacy for Cancer Patients. C-SUPPH1: Positive attitude. C-SUPPH2: Self-decompression. C-SUPPH3: Self-decision-making.

Based on these findings, an SEM was developed in Amos 26.0 to further test the proposed relationships. Variables that were significant in the univariate analysis, such as TNM stage and type of surgery, were included as control factors. Within the SEM, RHDS was defined as the predictor, FHI as the mediator, and C-SUPPH as the outcome variable. The standardized path coefficients are shown in Figure 3. All model fit indices reached the recommended criteria (Table 4), suggesting that the model adequately fit the data.

**Table 4:** Structural equation fitting index.

Index	χ2/df	RMSEA	GFI	NFI	IFI	TLI	CFI
Result	1.479	0.043	0.972	0.957	0.986	0.978	0.985
Standard value	<3.000	<0.080	>0.900	>0.900	>0.900	>0.900	>0.900

χ^2^/df, the ratio of chi-square to degrees of freedom; RMSEA, root mean square error of approximation; GFI, goodness-of-fit index; NFI, normed fit index; TLI, Tucker-Lewis index; CFI, comparative fit index; IFI incremental fit index.

A bootstrap analysis with 5,000 resamples was conducted to assess the mediation effect. The 95% confidence intervals for the direct, indirect, and total effects all excluded zero, indicating statistical significance. Results confirmed that FR acted as a partial mediator in the association between RHD and SME. Specifically, the overall effect of RHD on SME was 0.858, comprising a direct effect of 0.603 and an indirect effect of 0.254 via FR. Thus, the mediating pathway explained 29.60% of the total effect, highlighting the partial mediating role of FR (Table 5).

**Table 5:** Analysis of the mediating effect of family resilience between readiness for hospital discharge and self-management efficacy.

Effect	Effect size	Standard error	*p*	95% CI	Relative effect ratio (%)
Indirect effect	0.254	0.083	<0.001	0.128 ~ 0 0.467	29.60
Direct effect	0.603	0.182	<0.001	0.269 ~ 0.991	70.28
Total effect	0.858	0.183	<0.001	0.513 ~ 1.217	–

## 4 Discussion

This study aimed to explore the relationships among readiness for hospital discharge, family resilience, and self-management efficacy in BC patients, and further to examine whether family resilience mediates these associations.

The results showed that the mean score of RHDS was 85.56 ± 9.62, which was at a moderate level and consistent with the findings of Nowak et al. (2019). Among the subdimensions, coping ability scored the highest, followed by expected support, while personal status was the lowest. This suggests substantial differences in readiness for hospital discharge across different domains. A higher coping ability score indicates that most patients had acquired basic knowledge of disease management, medication adherence, and functional recovery. By contrast, the relatively lower score in expected support reflects uncertainty about whether sufficient medical resources, social support, and family assistance would be available after discharge. Such concerns may stem from insufficient information provision, lack of preparedness among spouses or family members, and patients’ limited understanding of rehabilitation pathways (Bristol et al., 2023). This aligns with the phenomenon of “discharge anxiety” described in prior studies, highlighting the importance of planning and post-discharge support continuity (Bockhorst et al., 2025). The lowest score in personal status suggests that many patients were still physically and psychologically vulnerable at discharge. This may be attributed to the significant side effects of chemotherapy (e.g., fatigue, nausea, alopecia) and incomplete postoperative functional recovery, which undermine patients’ confidence in life reconstruction and role resumption (Partridge et al., 2015).

The mean FHI score was 42.55 ± 5.70, indicating a moderate level, consistent with the findings reported by Wei et al. in colorectal cancer patients (Wei et al., 2024). This indicates that while most families demonstrated some degree of resilience in the face of cancer, challenges remained in coping, responsibility-sharing, and belief reconstruction (Tao et al., 2023). Unlike previous studies that emphasized individual resilience, the current study highlights the essential role of the family system as the foundation for illness adaptation. Families with higher resilience tend to exhibit stronger cohesion, clear goals, and proactive help-seeking behaviors, all of which contribute to a supportive caregiving environment. Prior research has shown that higher levels of family resilience are associated with better individual resilience, quality of life, and treatment adherence in cancer patients (Tao et al., 2023). However, in the context of Chinese culture, emotional suppression and overemphasis on caregiving duties may limit the full expression of family resilience (Huang et al., 2025). Some participants in this study reported that although family members were willing to help, difficulties remained in communication, emotional co-regulation, and shared decision-making. This suggests that clinical interventions should not only target individuals but also aim to enhance the adaptive capacity of the family system as a whole.

The mean C-SUPPH score was 95.62 ± 13.16, also at a moderate level, consistent with the findings of Zhuang et al. This indicates that while BC patients possessed some behavioral control and health responsibility, there remains considerable room for improvement. Higher scores in positive attitude and stress reduction suggest that most patients had psychological resources and emotional regulation abilities, such as cognitive reframing and self-motivation, to strengthen recovery confidence. This may be related to the increasing availability of psychological support, health education, and peer interventions (Jansen et al., 2023). In addition, the relatively high proportion of younger and middle-aged patients in this sample may contribute to greater adaptability and proactive emotional management. By contrast, the lowest score in self-decision-making reflects patients’ limited involvement in treatment choices, care behaviors, and rehabilitation planning, and their reliance on healthcare professionals (Liang et al., 2022). This finding, frequently observed in female cancer populations, may be explained by traditional physician–patient relationship models and barriers in accessing medical information, which restrict patients’ participation in key health decisions. Given the long recovery process and frequent complications of BC, professional care alone is insufficient to meet long-term individualized needs (Vilardaga et al., 2022). Self-management efficacy, therefore becomes a decisive factor in enabling patients to identify bodily signals, adjust lifestyle behaviors, respond to health changes, and actively engage in treatment. Thus, self-management efficacy is not only a psychological construct but also a behavioral foundation critical for long-term recovery and improved quality of life (Wu et al., 2020).

The SEM further supported Hypothesis 1, indicating that RHD was positively associated with FR (*r* = 0.408, *p* < 0.01), and FR was positively associated with SME (*r* = 0.386, *p* < 0.01). These findings suggest that RHD not only reflects physical stability or informational preparedness but may also indirectly enhance patients’ confidence and coping ability through reinforcing adaptive family dynamics. This is consistent with the findings of Li et al., who demonstrated that RHD significantly enhances patients’ self-care ability, reinforcing the concept that “discharge is not the end, but the starting point of healthy behavior” (Li et al., 2025).

More importantly, this study confirmed Hypothesis 2: FR partially mediated the relationship between RHD and SME, with the indirect effect accounting for 29.60% of the total effect. This suggests that even with adequate discharge preparation, patients may fail to transform readiness into sustainable health behaviors if their families lack cohesion, flexibility, or adaptive beliefs. In the context of BC, which heavily relies on family caregiving, patients need not only logistical and emotional support from family members but also shared meaning, joint responsibility, and coordinated problem-solving. Resilient families provide platforms for belief reconstruction, emotional buffering, and constructive adjustment, thereby strengthening patients’ engagement in long-term illness management (Huang et al., 2022).

Clinically, these results emphasize the necessity of integrating family-centered approaches into discharge planning. Health professionals should assess both the patient’s discharge readiness and family resilience before discharge, incorporating family members into education sessions and coping skills training. Tailored discharge plans could include structured communication guidance, shared goal-setting, and stress-management strategies designed for both patients and caregivers. Moreover, post-discharge follow-up should extend beyond physical recovery to include family adaptation and resilience reinforcement through community-based or digital family support programs.

From a research perspective, future longitudinal studies are warranted to examine how family resilience evolves and influences long-term recovery trajectories. Such studies could explore whether interventions enhancing family resilience produce sustained improvements in self-management efficacy and quality of life. Additionally, considering cultural and socioeconomic diversity, future work should assess whether these findings generalize to families from different social classes or cultural backgrounds. Including caregiver-related variables—such as caregiving burden, emotional support, or financial responsibility—would further clarify dyadic or family-level interactions influencing recovery outcomes.

## 5 Conclusion

The findings indicate that RHD significantly predicts SME, with FR partially mediating this relationship. This highlights the critical role of both individual readiness and FR in supporting recovery among BC patients.

### 5.1 Limitations

First, this was a single-center study using convenience sampling, which may limit the generalizability of the findings. Future research should include multi-center samples with broader demographic and regional representation. Second, data were collected only from patients, without incorporating perspectives from family members or caregivers, which restricted exploration of dyadic or family-level interactions. Future studies should include dyadic or family-based data collection. Third, the cross-sectional design precludes causal inference. Longitudinal or interventional research is needed to further validate the mechanisms underlying the role of family resilience in recovery.

## Data Availability

The original contributions presented in the study are included in the article/supplementary material, further inquiries can be directed to the corresponding authors.
